# Camouflage and Exploratory Avoidance of Newborn Cuttlefish under Warming and Acidification

**DOI:** 10.3390/biology11101394

**Published:** 2022-09-24

**Authors:** Mélanie Court, José Ricardo Paula, Marta Macau, Eve Otjacques, Tiago Repolho, Rui Rosa, Vanessa Madeira Lopes

**Affiliations:** 1 MARE—Marine and Environmental Sciences Centre & ARNET—Aquatic Research Network, Laboratório Marítimo da Guia, Faculdade de Ciências, Universidade de Lisboa, Av. Nossa Senhora do Cabo, 939, 2750-374 Cascais, Portugal; 2 Departamento de Biologia Animal, Faculdade de Ciências, Universidade de Lisboa, Campo Grande, 1749-016 Lisboa, Portugal; 3 Carnegie Institution for Science, Division of Biosphere Sciences and Engineering, Church Laboratory, California Institute of Technology, 1200 E. California Blvd, Pasadena, CA 91125, USA

**Keywords:** *Sepia officinalis*, cephalopod, embryogenesis, climate change, crypsis, disruptiveness, open-field

## Abstract

**Simple Summary:**

Sub-lethal effects of climate change on organisms have received little attention to date. For instance, little is known concerning the ability of cuttlefish to camouflage and to explore under ocean acidification and warming. This study aimed to evaluate the physiology, camouflage performance, and exploratory avoidance behavior of cuttlefish hatchlings exposed to these stressors during embryogenesis. Hatchlings were placed in arenas with either sand or white and black gravel covering the bottom. Photographs were taken remotely to extract camouflage latency and pixel values in the cuttlefish body and the background. Mobility and proximity to the arena walls were recorded. Despite survival being lower under acidification and warming combined, our results indicate that camouflage was strengthened under warming, whilst no effect was found on spatial exploration. This study shows that cuttlefish mobility and exploratory avoidance behaviors are unlikely to be impacted by changes in climate. Moreover, camouflage, an anti-predator strategy essential to the survival of cuttlefish newborns, is not impeded and might be enhanced by future levels of ocean acidification and warming.

**Abstract:**

Ocean warming and acidification have been shown to elicit deleterious effects on cephalopod mollusks, especially during early ontogeny, albeit effects on behavior remain largely unexplored. This study aimed to evaluate, for the first time, the effect of end-of-the-century projected levels of ocean warming (W; + 3 °C) and acidification (A; 980 µatm *p*CO_2_) on *Sepia officinalis* hatchlings’ exploratory behavior and ability to camouflage in different substrate complexities (sand and black and white gravel). Cuttlefish were recorded in open field tests, from which mobility and exploratory avoidance behavior data were obtained. Latency to camouflage was registered remotely, and pixel intensity of body planes and background gravel were extracted from photographs. Hatching success was lowered under A and W combined (AW; 72.7%) compared to control conditions (C; 98.8%). Motion-related behaviors were not affected by the treatments. AW delayed camouflage response in the gravel substrate compared to W alone. Moreover, cuttlefish exhibited a higher contrast and consequently a stronger disruptive pattern under W, with no changes in background matching. These findings suggest that, although climate change may elicit relevant physiological challenges to cuttlefish, camouflage and mobility of these mollusks are not undermined under the ocean of tomorrow.

## 1. Introduction

The world’s climate has undergone extensive changes since the pre-industrial era, a process described by Duarte [1] as Anthropogenic Global Change, i.e., “the global-scale changes resulting from the impact of human activity on the major processes that regulate the functioning of the Biosphere”. Jointly, warming, acidification, and deoxygenation are viewed as the “deadly trio” due to their central role in most historical global mass extinctions and their alarming importance in modern world change [2,3]. The rise in CO_2_ concentration in the atmosphere leads to ocean acidification (OA) [4] and ocean warming (OW) resulting from an increased greenhouse effect. Due to its high density and low specific heat, the ocean acts as a heat reservoir, and consequently buffers changes in climate [5]. Indeed, it is estimated that the ocean has absorbed 91% of the energy released by global warming between 1971 and 2018 [6], most of it accumulating in the upper 700 m of the ocean [7]. End-of-the-century sea surface temperature change projections range from stabilization at current temperatures to a ~5°C temperature increment [6], depending on the Shared Socioeconomic Pathway (SSP) and the global effective radiative forcing. The direct effects of Ocean Warming (OW) on organisms include increased metabolism costs, hindered oxygen delivery to tissues and consequent narrower ecological niche [8], and increased vulnerability to disease, which often results in mass mortalities [9,10,11]. Accordingly, fisheries are susceptible to a decrease in the maximum catch potential of many fish stocks, averaging a circa 4% decrease per decade [12]. Nevertheless, OW also induces lasting changes in communities through altered species interactions, such as changes in bottom-up forcing due to a predominance of resistant plankton species [13]. 

Similarly, owing to CO_2_’s high solubility in water, the ocean is estimated to have absorbed from 20% to 30% of anthropogenic CO_2_ emissions since the late 1980s, corresponding to a decrease in ocean pH of 0.1 units. According to the Intergovernmental Panel on Climate Change (IPCC), this drop is expected to persist, with pH declining as far as an additional 0.32 units by 2100 under a high emissions scenario (RCP8.5) [12], and an additional ~ 0.4 units if the SSP is accounted for, under fossil fuel-based development (SSP5-8.5) [14]. Ocean acidification has several detrimental effects on marine biota. With respect to the adverse impacts, it compromises the ability of certain organisms to develop skeletons, namely corals, calcareous plankton, and other calcium carbonate shell-forming groups [4]. Furthermore, elevated CO_2_ and subsequent acidification result in higher energetic costs for animals, as consumers require additional energy for acid-base regulation, ultimately affecting growth, survival, and reproduction [15]. Severe repercussions of OA are expected to also alter species interactions, for example, through increased competition from organisms that are more resilient to or benefit from OA [16,17]. Nevertheless, organismic responses to OA are often species-specific and vary according to the environment. Species in naturally low pH environments, e.g., hydrothermal vents, oxygen-minimum zones, and upwelling sites, or environments with high pH spatial and temporal variability, such as coastal zones and shelf-seas, seem less affected by OA. Yet, as they are often near their biological limits, these species might be exposed sooner to harmful thresholds [18]. 

Cephalopods were observed by Doubleday et al. [19] to have undergone a population expansion between 1953 and 2013, whilst most fish populations were declining. This phenomenon co-occurred with large-scale processes, such as overfishing-driven competition and predation relaxation, and anthropogenic climate change. Indeed, ocean warming leads to faster growth rates and shorter life cycles, providing a competitive edge relative to longer-lived species [19]. Like most cephalopods, *Sepia officinalis* are highly adaptable to environmental conditions, considering their high fecundity and short life span [20,21]. Tolerance to climate-related changes is likely to differ between juveniles and adult cuttlefish, which can reach 310 mm in mantle length [22]. On the one hand, hatchlings must allocate considerable energy to rapid growth. On the other, they may have exhausted their maternal yolk supply and must contend with high food intake requirements due to a faster metabolism [23,24]. 

Cuttlefish embryonic stages were found to have lower survival rates and higher premature hatching under acidification and warming combined (+4 °C, Δ0.5 pH, ~1600 μatm CO_2_), as well as under warming alone [25]. Further, pre-hatching eggs display lower hypoxic thresholds when exposed to both stressors simultaneously (*S. officinalis*) [25]. However, acidification per se was found to have no impacts on cuttlefish hatchlings’ fitness, i.e., ability to reproduce [25,26], except for increased rates of calcification in the cuttlebone at 980 μatm (assuming cuttlefish are fed) [27], or starting from a decrease of 0.25 pH units [28] relative to current pH levels. Concerning behavior, Moura et al. [26] observed no effects of acidification on shelter-seeking, hunting, or detection of conspecifics in cuttlefish hatchlings. To date, the effects of warming on cephalopod behavior are unknown.

Animal movement tracking in a laboratory setting is commonly used to construe the animal’s foraging and anti-predator behaviors [29]. This method is useful to study the impact of climate changes on the movement of cuttlefish newborns, and therefore potential impacts on their foraging success. In addition, it allows us to determine whether cuttlefish exhibit thigmotactic behaviors, as the tendency to remain close to vertical surfaces is viewed as an attempt to take cover from predators. Thus, venturing into an open space, especially when accompanied by energy and time costs, represents a voluntary, curiosity-driven movement, often motivated by mating or hunger [30]. This exploratory behavior, a movement directed toward acquiring information about the environment, is evoked in animals of all phyla [31], and can, when inhibited, limit foraging opportunities. 

Conversely, camouflage in the European cuttlefish has been extensively studied. They are uniquely equipped to dynamically camouflage, as they control their skin coloration and texture neurologically, based solely on their vision [32]. Apart from exhibiting an impressive repertoire of skin patterns and a sophisticated visual system, this characteristic allows them to change skin patterning instantly [32,33]. Despite being color-blind [34], cuttlefish are able to closely emulate background colors [35] and are particularly responsive to edges and differences in contrast [36,37]. Although this species displays a continuum of chromatic body patterns, three types are commonly recognizable based on the size of light and dark spots. It produces uniform (small spots) and mottle (medium-sized spots) patterns to match the background and hinder detection [32,36,38] and disruptive patterns (large spots, and other forms) in order to prevent recognition by creating the appearance of false edges and boundaries and hiding the animal’s true outline and shape [39]. Camouflage in juveniles is crucial for survival, as predation rates are high. Whilst adult cuttlefish use body patterning mostly in reproductive behaviors, juveniles use it primarily for concealment, wherein light mottle and disruptive patterns are most useful [32]. 

Sub-lethal effects of climate change on organisms should be considered when attempting to predict ecosystem changes, particularly for organisms occupying a central position in the food web, such as the cuttlefish. The aim of this study was to evaluate, for the first time, the effect of end-of-the-century projected levels of ocean warming (+ 3 °C; SSP2-4.5) and acidification (980 µatm *p*CO_2_; SSP3-7.0) on *S. officinalis* early development, exploratory avoidance behavior and ability to camouflage in different substrate complexities (sand and black and white gravel). More specifically, we investigated embryonic development time, hatching success, exploratory behavior (proximity of the novel object, acceleration and immobility rate) and ability to camouflage (latency, disruptiveness, background matching) under the different climate change-related treatments.

## 2. Materials and Methods

### 2.1. Ethical Statement

All experiments were approved by the FCUL Animal Welfare Committee (ORBEA FCUL) and the Portuguese General-Directorate for Food and Veterinarian Contacts (DGAV), in accordance with National (Decreto-Lei 113/2013) and EU legislation (Directive 2010/63/EU) on the protection of animals used for scientific purposes. The number of animals used in this study was reduced to circa 80 per treatment, enough to ensure that differences between treatments were detected. After the experiments, animals were anesthetized following the recommended guidelines for animal welfare [40] and the tissues fixated to posteriorly analyze the brain chemistry of animals exposed to these climate-change-related stressors. The prospective severity of the procedures used in this experiment was assessed according to Cooke et al. [41] as between sub-threshold (behavioral trials) and moderate (exposure to physiological stress—acidification and warming). The severity estimation for the sacrifice of animals is non-recovery. The retrospective severity of these procedures on cuttlefish hatchlings is consistent with that which was previously evaluated.

### 2.2. Egg Collection and Husbandry

*S. officinalis* egg clutches (4) at early development stages were collected off Algés and Cascais, Portugal, by local fishermen, in May 2021 and transferred to the Laboratório Marítimo da Guia (Cascais). The eggs (*n* = 248) were separated from the clutches and placed in two semi-opened recirculating aquaria systems, each comprising two water-baths (four water-baths in total) and a sump. Each system, corresponding to one treatment, contained four 9-L plastic tanks (replicates), each connected to the bath through two small meshes, and receiving water directly from the sump (Figure S1). The eggs were distributed randomly and in equal number (within the treatment) between the tanks, acclimated during three days at control conditions and reared at (i) control (18 °C, *p*CO_2_ = 420 µatm, *n* = 80); (ii) warming (21 °C, *p*CO_2_ = 420 µatm, *n* = 80); (iii) acidification (18 °C, *p*CO_2_ = 980 µatm, *n* = 80) and (iv) warming and acidification combined (21 °C, *p*CO_2_ = 980 µatm, *n* = 88; as fewer successful hatchings are expected in this treatment). Water was pumped directly from the sea and filtered through a 1-µm mesh and sterilized by a 12-W UV-sterilizer (Vecton 120 Nano, TMC-Iberia, Lisbon, Portugal). Water was continuously renewed with a water drip system in each bath. Each system was connected to a 270-L sump by 50-W pumps (TMC, V2 Power Pump, 3000 L h^−1^), containing a protein skimmer (ReefSkimPro 400, TMC-Iberia, Lisbon, Portugal) and bioballs (ouriço^®^, Fernando Ribeiro Lda, Queluz, Portugal). Additionally, one 35-W pump (TMC, V2 Power Pump, 2150 L h^−1^) was connected to each bath, at 288–390 mL min^−1^ flow rate in each tank (renewal every ~30 min). The tanks were illuminated from above with LED, 8-W lights, under a photoperiod of 14 h light to 10 h dark, in accordance with the concurrent local diurnal cycle. Water was kept oxygenated using eight air stones, two in each sump, and one in each bath connected to an air compressor (Medo Blower LA-120A, Nitto Kohki, Tokyo, Japan, sourced from UK branch, Derbyshire). Temperature was maintained through a temperature controller (XH-W3002, accuracy ± 0.1 °C, hysteresis 0.3 °C) connected to water heaters (Eheim thermocontrol 150, Eheim GmbH & Co KG, Deizisau, Germany) and a water chiller (Hailea HC-150A). Via solenoid valves, pH was adjusted automatically, regulated by a Profilux controlling system (3N GHL, Kaiserslautern, Germany) connected to two pH probes (VWR, double junction epoxy BNC, Darmstadt, Germany, sourced through Avantor, Carnaxide, Portugal, hysteresis 0.05). pH values were read every 2 s and downregulated by injection of a certified CO_2_ gas mixture (Air Liquide, Algés, Portugal) through air stones, or upregulated by aerating the tanks with CO_2_-filtered air, using soda lime (Sigma-Aldrich, Darmstadt, Germany). Alkalinity was tested thrice a week with a digital titrator (Sulfuric Acid 0.1600 N; Hach, Loveland, CO, USA) in order to adjust the pH corresponding to 980 µatm of *p*CO_2_ through the CO2SYS Program 01.05 [42]. Salinity (Hanna refractometer, accuracy ± 1 PSU), oxygen levels and temperature (oximeter VWR DO220, accuracy ± 1.5%, ± 0.3 °C respectively), and pH (pHmeter VWR pHenomenal, accuracy ± 0.005) were monitored daily (Table S1). Ammonia/ammonium, nitrite, and nitrate levels were monitored every week through saltwater colorimetric tests (TropicMarin, Hünenberg, Switzerland) and maintained below 0.02 mg L^−1^ (nitrites and ammonia/ammonium: accuracies ± 0.02 mg L^−1^ and ± 0.03 mg L^−1^, respectively) and 0.5 mg L^−1^ (nitrates: accuracy ± 0.5 mg L^−1^). 

### 2.3. Hatching Success, Development Time and Size

Mantle length was inferred from novel object test videos in ImageJ 1.46r (National Institute of Health, Bethesda, MD, USA), compared to a two-cent coin (EUR). Hatching success (number of hatchlings divided by the number of eggs) and development duration from the time the eggs were collected were registered upon hatching. After five days with no hatching, in all treatments, hatchings were considered unsuccessful. To discern individual specimens, newly-hatched cuttlefish were placed in plastic cups (labeled with cuttlefish number, treatment, replicate tank, and hatching date) with eight mesh-covered openings for water circulation, within their rearing tank.

### 2.4. Exploratory Avoidance Behavior Data Collection and Processing

Exploratory avoidance behavior was assessed through an open-field test with a novel object in the center [43]. From two to five days post-hatching (before the cuttlefish require feeding), 180 cuttlefish (45 per treatment), were placed with a black spoon in a white circular arena (12 cm diameter), with a purple bottle cap in the center (novel object), previously filled with 400 mL of water from the respective treatment (Figure S2). Black flaps surrounded the arena and prevented cuttlefish from seeing the observer. The light was directed above and reflected throughout the chamber with white styrofoam to diffuse it. A video camera (LEGRIA HF R56, 35 Mbps, Canon, Porto Salvo, Portugal) recorded the arena from above at a ca. 90° angle for 20 min upon placing the cuttlefish in the arena. From video recordings, cuttlefish movements were tracked using the animal tracking software ToxTrac v2.61 (see [44]), via the algorithm ToxId [45]. The detection rate (proportion of the video recording wherein the animal was detected by the software), average acceleration, immobility rate and duration of staying away from the walls and near the object (time spent in the region of interest—a circle [7 cm diameter] that is slightly elevated, thus disincentivizing exploration behavior), and inking events were extracted from videos. Video contrast was augmented beforehand by 130–150%. Results were considered exclusively when detection rates exceeded 90%.

### 2.5. Camouflage Data Collection and Processing

From two to five days post-hatching, and at least five hours after the open-field trial, 160 cuttlefish (40 per treatment) were placed, with the aid of a black spoon, in a white circular arena (12 cm diameter), with the bottom covered in either sand, to evoke a mottle pattern in the cuttlefish, or a gravel mixture of 60% black, 40% white (FishPlanet, Lisbon, Portugal), to evoke a disruptive pattern. Individual gravel area ranged from approximately 100% to 200% of the cuttlefish’s dorsal square area—a light region expressed under the disruptive pattern in the center of the mantle. The order of presentation of substrate patterns alternated between each trial, i.e., if a cuttlefish underwent the camouflage trial in sand first, and gravel immediately after, the following cuttlefish was placed in gravel first, then sand. Cuttlefish were attributed a random number displayed in videos and the observer was blind during the analysis, thus avoiding observer bias. The arenas were filled with water (400 mL) from the corresponding treatment and renewed between each trial. To register attempts at burying in the substrate, a video camera (GoPro Hero 3+, San Mateo CA, USA) recorded the arena for ten minutes after acclimation (considered when the cuttlefish remained stationary for more than five seconds). Concomitantly, photographs (Canon PowerShot G7X Mark II, white balance-calibrated, shutter speed 1/15, F-stop f/11, ISO 250, 1080p, 60 fps) were taken remotely (Canon Connect application for mobile phones) at a 90° angle approx., whenever cuttlefish changed camouflage pattern or intensified the present pattern. Time past acclimation was registered with the aid of a chronometer, upon taking each photograph. 

Camouflage latency was assumed as the time following acclimation until the photo was taken, when cuttlefish camouflage was best suited to the environment (in sand, strong mottle; in gravel, dark uniform, or strong disruptive pattern). Due to latency data being highly zero-inflated, it was transformed to binary data (immediate—from 0 to 30 s, and delayed camouflage—upwards of 30 s). Further, the difference between minimum and maximum pixel values (grayscale) within the frontal and transversal body planes, and the difference in pixel integrated densities (grayscale) between the cuttlefish’s light region (dorsal square) and white substrate and dark region and black substrate (preference was given to gravel within the cuttlefish’s field of vision) were extracted from photographs taken in gravel substrate through the ImageJ software (Figure S3).

### 2.6. Anesthesia and Humane Killing

After the open-field test, cuttlefish were carefully transferred to 50-mL Falcon tubes containing water from their treatment bath and 2% ethyl alcohol (EtOH). After 10 min, they were placed in 4%-EtOH Falcons, where they remained for another 10 min. They were then examined for mantle and syphon contractions and the mantle was pinched to detect responses to noxious stimuli [40] (all were unresponsive). As their brains were needed for further analyses, their death was confirmed by a knife incision between the head and the mantle. The mantles were immediately frozen at −80°C, and the head tissue was fixated for posterior neuron quantification analyses.

### 2.7. Data Analyses

#### 2.7.1. Survival Analysis

Hatching of cuttlefish over time was assessed through a Cox proportional hazards regression model (function “coxph”, package “survival”), with development time and successful hatchings (binary factor) as covariates and treatment as the predictor variable (four-level factor) (see [46]). The assumptions of the “coxph” model (proportional hazards, no over-influential observations and linearity of covariates) were tested by plotting the scaled residuals over time (Schoenfeld test; “ggcoxzph”). Since these were not met, a non-parametric “survdiff” model was fitted. Post-hoc multiple comparisons were performed, and *p*-values were adjusted through Bonferroni–Hochberg corrections, to avoid type I errors. 

#### 2.7.2. Generalized Linear Models

A Linear Model (LM) was fitted to mantle lengths, with treatment as the predictor variable.

Generalized Linear Models (GLMs) from the Beta family (log link function; function “betareg”, package “betareg”) were fitted to the percentage of time in the proximity of the object and immobility rate and an LM was fitted to average acceleration. The AIC (Akaike Information Criterion) function was used to determine whether the replicate, detection rate, mantle length and their interactions influenced the response variables.

LMs were used to assess pixel value differences between treatments (in the cuttlefish’s body planes and in comparison with the background). GLMs from the Binomial family (logit link function) were fitted to latency to camouflage, time of acclimation, burial in sand. The influence of replicates, first substrate presented, cuttlefish mantle length, and time elapsed between trials (and their interactions), was tested through the AIC function for each response variable. All GLM assumptions (independence, normality, and homoscedasticity of residuals) were tested. Type II Wald chi-squared tests (function “Anova”) were performed before each analysis to assess the influence of explanatory variables (treatment and first substrate presented) on the response variable. Post-hoc comparisons between treatments were performed (function “emmeans”, package “emmeans”). In order to avoid type I errors, *p*-values were adjusted through Tukey corrections. The admissible error was set at 0.05. Analyses were carried out in the RStudio, version 1.4.1717, PBC, software.

## 3. Results

### 3.1. Development Time, Hatching Success and Size

Cuttlefish exhibited a prolonged embryogenesis under Acidification (A; ~50 days, *n* = 50) relative to control conditions (~46 days; Cox model, *n* = 51, *p* < 0.001) (Table 1; post-hoc tests shown in Table S2), while Warming (W) caused cuttlefish to hatch sooner (~ 35 days; Cox model, *n* = 49, *p* < 0.001). Such effects were reduced under Acidification and Warming combined (AW; ~37 days; *n* = 55, *p* = 0.03).

**Figure 1:**
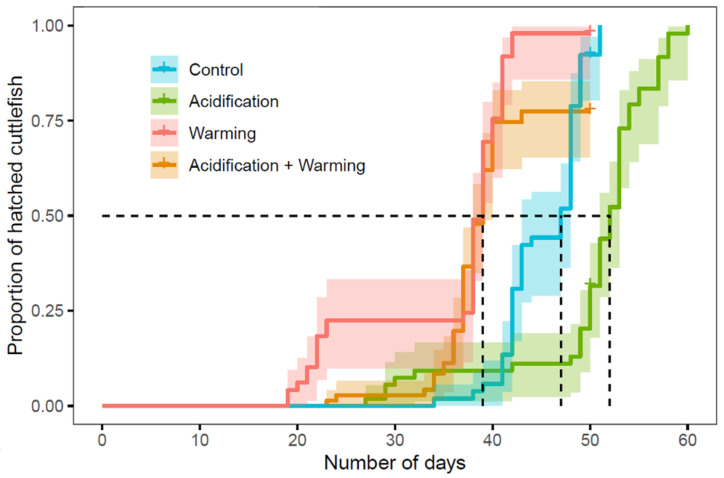
Proportion of cuttlefish that hatched over time (from the start of acclimation, integrating development time and hatching success), related to different treatments: control, acidification, warming, and acidification and warming. The dotted lines indicate the day at which 50% of cuttlefish were born in each treatment.

The AW treatment decreased hatching success to 72.7% relative to control (98.8%), A (95.0%) and W (98.8%) conditions (Figure 1). Although no statistically significant effect of A (LM, z = 0.928, df = 167, *p* = 0.790) and W (LM, z = −2.508, df = 167, *p* = 0.059) on mantle length was detected compared to C conditions, AW-exposed cuttlefish presented reduced mantle length relative to W-exposed cuttlefish (LM, z = −3.33, df = 167, *p* < 0.01).

### 3.2. Exploration Avoidance 

Exploratory avoidance and locomotory behaviors were inferred through the acceleration, immobility and distancing from the arena walls (proximity to the object) in an open-field test. Results are shown in Table 2. No significant differences on average acceleration (Wald chi-squared test, df = 3, χ^2^ = 1.35, *p* > 0.1) were observed among treatments (Figure S4). 

Moreover, the time spent near the object was also not affected by the different treatments (Wald chi-squared test, df = 3, χ^2^ = 3.18, *p* > 0.1) (Figure S5), nor were ink ejections (Wald chi-squared test, df = 3, χ^2^ = 3.09, *p* > 0.1).

### 3.3. Camouflage

The ability to camouflage was assessed through the latency to camouflage, the intensity of the disruptive pattern and the matching to the background (comparison of pixel intensities) (statistical outputs are shown in Table 3, post-hoc comparisons in Table S2). 

**Figure 2:**
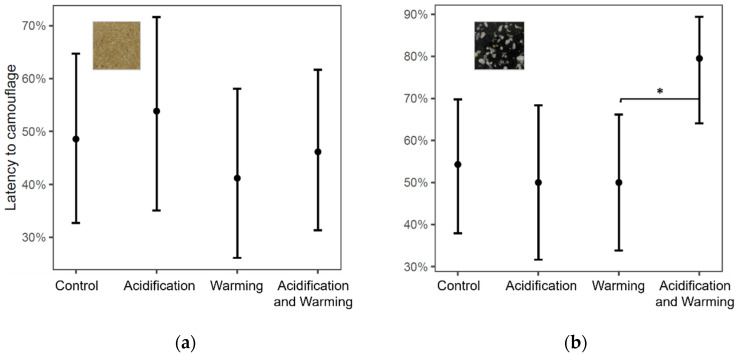
Proportion of cuttlefish exhibiting delayed camouflage in (**a**) sand; (**b**) gravel substrates, with relation to different treatments: control, acidification, warming, and acidification and warming. Points represent predicted means, and bars represent confidence intervals from generalized linear models (Binomial family). *: *p* < 0.05).

Latency to camouflage on the sand substrate did not change among treatments (Wald chi-squared test, df = 3, χ^2^ = 1.03, *p* > 0.1; Figure 2). Yet, fewer cuttlefish camouflaged immediately upon acclimation in the gravel under AW relative to W (GLM, z = 2.15, df = 167, *p* < 0.05). Camouflage in gravel was delayed when the gravel substrate was presented first (Wald chi-squared test, df = 1, χ^2^ = 5.65, *p* < 0.05).

**Figure 3:**
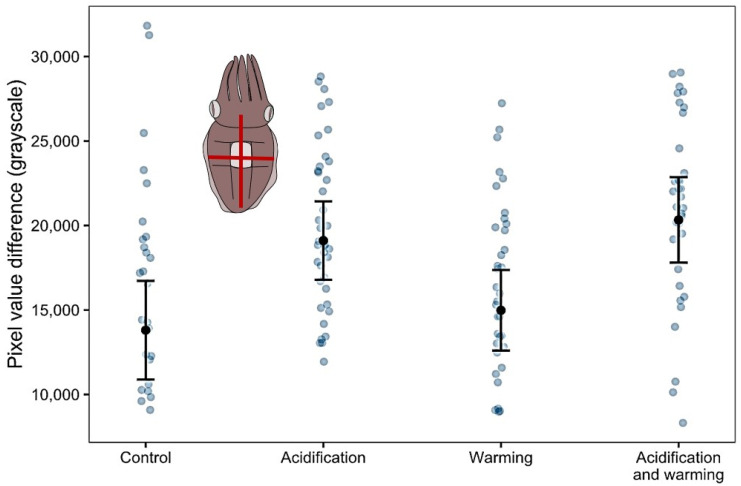
Pixel values range (difference between the maximum and minimum pixel intensity; grayscale units) along the frontal and transversal body planes of cuttlefish exposed to treatments: control, acidification, warming, and acidification and warming combined. Points represent predicted means, and bars represent confidence intervals from a linear model. Blue points represent observed individual data.

With respect to body planes pixel intensity differences in gravel, both W (LM, z = −3.48, df = 134, *p* < 0.01) and AW (LM, z = 2.73, df = 134, *p* < 0.05) treatments significantly enhanced body pattern contrast compared with C (Figure 3). Moreover, body contrast was reduced when gravel was presented first (Wald chi-squared test, df = 1, χ^2^ = 8.72, *p* < 0.01).

Treatments did not affect the difference between the cuttlefish’s dark region and black gravel integrated pixel intensities (Wald chi-squared test, df = 3, χ^2^ = 4.92, *p* > 0.1) nor the difference between the cuttlefish’s light region (dorsal square) and white gravel integrated pixel intensities (Wald chi-squared test, df = 3, χ^2^ = 2.01, *p* > 0.1) (Figure S6). Pixel integrated density of the dark region did not fall lower than black gravel. 

Latency to camouflage (immediate or delayed) was lower in sand (GLM, df = 1, χ^2^ = 3.95, *p* = 0.05) (Figure S7). Indeed, more cuttlefish were able to camouflage immediately in the sand (52%) than in gravel (40%). Time of acclimation did not vary significantly among treatments (Wald chi-squared test, df = 3; sand, χ^2^ = 2.59, *p* > 0.1; gravel, χ^2^ = 1.00, *p* > 0.1), nor did attempts at burying in the sand (Wald chi-squared test, df = 3, χ^2^ = 2.59, *p* > 0.1).

## 4. Discussion

To evaluate possible sub-lethal effects of expected climate changes on a cephalopod species, we have studied exploratory and anti-predator behaviors of *Sepia officinalis* hatchlings. We have found through open-field and camouflage tests that this species, despite exhibiting pronounced mortality during its embryonic development, is highly resistant with respect to behavior.

As previously reported [25,26,28] for lower pH (Δ pH 0.4, 0.5, and 0.4, respectively), acidification of Δ pH ~0.3 had no relevant effect on hatching success. CO_2_ partial pressures can be three times higher within the egg than surrounding seawater *p*CO_2_ [25,28], making them uniquely prepared for projected levels of OA. Moreover, cuttlefish are active swimmers and consequently need an efficient ion transport system to maintain a stable blood pH during exercise in order to cope with their respiratory CO_2_ [47]. Indeed, contrarily to most marine invertebrates, ectotherms with high metabolic rates, such as teleosts and cephalopods, are able to garner soft tissue mass and calcify under hypercapnia [26]. Additionally, as observed by Moura et al. [26], exposure to A during embryogenesis was found to not affect the size of newly-hatched cuttlefish. Similarly to squid under Δ pH 0.4 [48] and cuttlefish under 980 µatm *p*CO_2_ [27], our findings indicate that acidification prolongs embryogenesis. Thus, on the one hand, A may pose a physiological burden on cuttlefish hatchlings if paired with the downregulation of regulatory and metabolic genes [49]. Warming, on the other hand, led to shortened development times as a result of higher energy expenditures and turnover [25,50]. Yet, acidification and warming were found to have a synergistic negative effect on hatching success, reducing it substantially. This is corroborated by Rosa et al. [25], with more extreme conditions (+4 °C, ΔpH 0.5). However, Dorey et al. [28] found no effect (+3 °C, ΔpH 0.4), which might suggest intraspecific differences in heat and hypercapnia tolerances associated with local adaptation. The combined treatment (AW) did not affect cuttlefish size (mantle length) at hatching, possibly due to the existence of an antagonistic effect between stressors, or because cuttlefish have developed mechanisms to temporarily cope with these stressors, such as improved systemic oxygen delivery through cellular and mitochondrial regulation [51]. Interestingly, AW-exposed cuttlefish displayed intermediate development durations between A and W, suggesting that acidification might reduce the negative effects caused by warming.

Exploratory behavior is the spatial exploration of novel situations; thus, inhibited exploratory behavior can potentially limit foraging opportunities. In the present case, the time cuttlefish spent in the proximity of the novel object was not affected by the treatments. This was also expected, as A does not affect shelter-seeking and hunting behaviors [26]. Similarly, average acceleration and immobility rate were not affected by climate change-related stressors (W, A, and combined). Accordingly, Maneja et al. [52] found that cuttlefish’s ability to capture prey was only affected at 4000 µatm *p*CO_2_, a very distant value from projected levels for the end of the century. Other pre-natal sources of stress, such as predator cues, were found to have no effect on locomotor activity [53]. Defensive behaviors, e.g., approaching, retreating, and inking in the presence of a predator, were not influenced by embryonic exposure to predator cues in *Sepia pharaonis* and *S. officinalis* [53]. However, O’Brien et al. [54] observed that embryonic stress (odor cues from predators and artificial lighting) increased attempts at capturing prey. According to these authors, locomotor activity levels did not change as a result of embryonic stress, but increased from maternal stress (daily removal of the reproducing mother from water). As per the threat-sensitive predator-avoidance hypothesis [55], animals exhibit antipredator behavior proportionately to the perceived threat from a predator. Indeed, antipredator behaviors are costly as they reduce foraging and mating opportunities. Animals that can accurately assess predation risk and adjust their behavior accordingly have a better chance at survival. Concurrently, a shift in activity levels would likely impact survivorship. Such results could be explained by a broad phenotypic plasticity, characteristic of cephalopods [56], paired with frequent exposure to extreme conditions. For example, species in high pH spatial and temporal variability environments, such as coastal zones and shelf-seas, seem less affected by A [18]. However, the effects of prolonged exposure into adulthood to these stressors are unknown.

Cuttlefish is well adapted to sandy substrates, as it camouflaged more promptly in this substrate independently of the different climate change-related treatments. In addition to chromatic patterns, texture patterns are equally necessary to match a sand background. Cuttlefish which did not exhibit skin texture, through the expression of major lateral papillae, i.e., protrusions borne of muscle expansions [33], could not successfully blend in the sand. Furthermore, cuttlefish seem to perform more poorly if they are first presented with another substrate. Indeed, camouflage was delayed, body contrast in gravel was reduced, and cuttlefish displayed darker colors when gravel was the first substrate presented to cuttlefish. A possible reason for this is increased stress at being in a new environment where burial was less facilitated. For instance, Allen et al. [57] found that *S. officinalis* show no preference for a particular substrate, save when they can bury themselves. 

In white and black gravel, a more complex substrate, the combined effect of A and W delayed cuttlefish camouflage relative to W per se. The optic lobe undergoes rapid development during embryogenesis, specifically structural components of the cortex and radial column zone, which are responsible for the processing of visual information [58]. Increased latency to disruptive camouflage might indicate that this development was compromised, due to an exacerbation of extreme conditions inside the egg (high *p*CO_2_, low O_2_), especially in late embryogenesis. Further, color change is likely to entail energetic or metabolic costs related to the synthesis of pigments and cells, or changes in the state of chromatophore cells [59]. Therefore, these processes might be impeded by the existing higher energy expenditure caused by warming. For example, guppies (*Poecilia reticulata*) were found to increase food consumption following color change and avoid further color changes [60], and *S. officinalis* L. intensified camouflage patterns only when exposed to visual [61] and small predators [62].

Given that camouflage in cuttlefish hatchlings is primarily a defense strategy, camouflage performance must be evaluated from the perspective of relevant predators [63]. Putative cuttlefish predators (di- and tri-chromatic fish) have been shown to rely minimally on color [64], which is why grayscale is considered a reliable measure of camouflage efficiency. Contrast in disruptive patterning is thus another indicator of camouflage performance, considering that cuttlefish adjust their contrast proportionately to the contrast of the background [65]. Interestingly, W, and, to a lesser extent, AW, enhanced body pattern contrast along the frontal and transversal body planes. Cuttlefish seemingly camouflage better when exposed to a stressor during embryonic development, or even when their progenitor is subjected to stress. Maternal stress (daily removal of the reproducing female from the water) was found to increase disruption in offspring [54]. Furthermore, embryonic exposure to predator chemical cues led to strengthened disruptive patterns in *S. officinalis* [63]. *S. officinalis* hatchlings face a higher predatory threat than embryos or adults [53]. However, their predators are expected to perish under future ocean conditions and overfishing [19]. This suggests that cuttlefish might waste resources by intensifying an anti-predator behavior in the absence of predators. However, these cuttlefish did not show improved background matching. This could be due to cuttlefish resorting to dark uniform patterns instead of the expected disruptive pattern in black and white gravel. It is important to note that gravel size was not controlled (cuttlefish only produce disruptive patterns if white gravel measures around 40% to 120% of their dorsal square [66]). 

Success of hatching reflected a synergistic effect of temperature and acidity. This might indicate that a threshold of extreme conditions was reached, triggering energy allocation toward behaviors conducive to foraging and protection from predators (where no interacting effect was found) and penalizing growth and survival. Thus, although no effect was detected on behavior, its damaging effects on physiology cannot be disregarded. 

## 5. Conclusions

Cuttlefish seem to be particularly resilient to acidification, a stressor that solely prolonged embryogenesis. However, warming had direct impacts on cuttlefish growth, leading to shortened development times and higher mortality. The combined effect of these stressors markedly reduced hatching success, denoting an interacting effect. No effect of acidification or warming on hatchlings’ exploration behavior and activity levels was identified in this study. Conversely, disruptive camouflage was enhanced under warming. This demonstrates some capacity to withstand and adapt to change, similarly to adaptations to predator exposure, or even artificial stressors (e.g., LED lighting), which have never been encountered by the species in its natural environment [54]. However, maximum disruptive camouflage was delayed by acidification and warming combined in relation to warming alone, suggesting an underlying physiological or developmental burden of camouflage. If climate change were to hamper camouflage considerably, cuttlefish would lose their primary defense strategy against predators. The present findings increase our understanding on the biological impacts of these climate change-related stressors on cephalopods. However, impacts of deoxygenation, hypoxia, and acute events such as marine heatwaves merit further investigation. Concurrently, as shown by O’Brien et al. [54], maternal stressors have a larger impact on post-natal behaviors than direct embryonic stress. Thus, studying the impacts of combined climate change stressors on reproductive females and their offspring might provide valuable insights into an integrated climate change response. Furthermore, these impacts on gametes, zygotes, and early development should also be understood, as these stages lack the specialized ion-regulatory epithelia that enable resilience to ocean acidification [67].

## Figures and Tables

**Table 1:** Results from statistical analyses, depicting the effect of the treatments (control, acidification, warming, and acidification and warming combined) on the physiology of *Sepia officinalis*.

Model	Response	Predictor	χ^2^	d.f.	*p*-Value
LM, identity link	Mantle length	Treatment	12.08	3	**0.0071**
Cox model	Hatching over time	Treatment	104	3	**<2 × 10^−16^**

*p*-values in bold are inferior to 0.05.

**Table 2:** Results from statistical analyses, depicting the effect of the treatments (control, acidification, warming, and acidification and warming combined) on locomotory and exploratory behaviors of *Sepia officinalis*.

Model	Response	*n*	Predictor	χ^2^	d.f.	*p*-Value
GLM, beta, log link	Proximity to the object	140	Treatment	3.18	3	0.3649
LM, identity link	Average acceleration	142	Treatment	1.35	3	0.7162
			Visibility rate	24.16	1	**8.8 × 10^−7^**
			Visibility rate: Treatment	7.89	3	**0.0484**
GLM, binomial, logit link	Ink ejection	158	Visibility rate	0.31828	1	0.5726
			Treatment	0.80615	3	0.8480
			Visibility rate: Treatment	0.17741	3	0.9812

*p*-values in bold are inferior to 0.05.

**Table 3:** Results from statistical analyses, depicting the effect of the treatments (control, acidification, warming, and acidification and warming combined) on the ability to camouflage of *Sepia officinalis*.

Model	Response	*n*	Predictor	χ^2^	d.f.	*p*-Value
GLM, binomial, logit link	Latency to camouflage (gravel)	134	First substrate	4.29	1	**0.0383**
			Replicate	2.44	3	0.4867
			Treatment	10.34	3	**0.0159**
			First substrate:Treatment	19.46	3	**0.0002**
			Replicate:Treatment	17.40	9	**0.0428**
GLM, binomial, logit link	Latency to camouflage (sand)	134	First substrate	6.69	1	**0.0097**
			Treatment	1.01	3	0.7984
			First substrate:Treatment	1.27	3	0.7352
LM, identity link	Pixel value difference in body planes	134	First substrate	8.74	1	**0.0032**
			Treatment	17.71	3	**0.0005**
			First substrate:Treatment	3.47	3	0.3243
LM, identity link	Pixel integrated density light region-white gravel	134	Treatment	2.13	3	0.5453
LM, identity link GLM, binomial, logit link	Pixel integrated density dark region-black gravel	134	Treatment	4.92	3	0.1776
			Replicate	7.96	3	**0.0476**
			Treatment	2.36	3	0.5018
GLM, binomial, logit link	Acclimation (sand)	126	Treatment	2.59	3	0.4587
GLM, binomial, logit link	Acclimation (gravel)	126	First substrate	12.26	1	**0.0004**
GLM, binomial, logit link	Burial in sand	134	Treatment	1.03	3	0.7948
			First substrate:Treatment	5.81	3	0.1215
GLM, binomial, logit link	Acclimation	252	Substrate	0.46	1	0.4998
GLM, binomial, logit link	Latency to camouflage	268	Substrate	3.95	1	**0.0469**

*p*-values in bold are inferior to 0.05.

## Data Availability

The data presented in this study are openly available in FigShare at 10.6084/m9.figshare.19214337, accessed on 25 August 2022.

## Supplementary Materials

The following supporting information can be downloaded at: https://www.mdpi.com/article/10.3390/biology11101394/s1, Figure S1. Aquaria system: (a) Recirculating system comprising two aquaria with two water-baths and a sump each, (b) four tanks in a bath (corresponding to a treatment); (c) cuttlefish eggs in a suspended net and cup containing a newborn cuttlefish. Figure S2: Photograph from a novel object test video frame. The purple cap is the novel object, and the green circle delineates the region of interest (in proximity of the object and slightly elevated). Figure S3. Metrics used to infer pixel intensity differences from photographs in the ImageJ software: (a) Cuttlefish transversal plane and (b) frontal plane used to measure pixel intensities; comparison of pixel integrated densities between (c) white substrate and cuttlefish light region and (d) black substrate and cuttlefish dark region. Figure S4. Effect of treatments (control, acidification, warming, and acidification and warming) on cuttlefish: (a) Average acceleration; (b) Percentage of time spent immobile. Points represent predicted means, and bars represent confidence intervals from generalized linear models (Gaussian and Beta families, respectively). Figure S5: Effect of treatments (control, acidification, warming, and acidification and warming) on the percentage of time cuttlefish spent in proximity of the novel object. Points represent predicted means, and bars represent confidence intervals from a generalized linear model (Beta family). Blue points represent observed individual data. Figure S6. Pixel values range (difference between the maximum and minimum pixel intensity; grayscale units) between: (a) Black gravel and the cuttlefish’s dark region; (b) White gravel and the cuttlefish’s light region (dorsal square) of cuttlefish, with relation to treatments, viz., control, acidification, warming and acidification and warming combined. Points represent predicted means, and bars represent confidence intervals from linear models. Blue points represent observed individual data. Figure S7: Influence of substrate (white and black gravel or sand) on the proportion of cuttlefish exhibiting delayed camouflage. Points represent predicted means, and whiskers represent confidence intervals from generalized linear models (Binomial and Poisson families, respectively). Table S1: Seawater parameter values measured daily in each tank during exposure to treatments, shown as mean ± standard deviation. TA—Total Alkalinity, TCO_2_—Total CO_2_, HCO_3_—Bicarbonate, ΩAr—Aragonite saturation state. Table S2: Results from post-hoc multiple comparisons, depicting the effect of the treatments (control, acidification, warming, and acidification and warming combined) on the physiology, camouflage ability and locomotor parameters of *Sepia officinalis*.
