# Supplementary material for: Camouflage and Exploratory Avoidance of Newborn Cuttlefish under Warming and Acidification
Source: Biology (Basel). 2022 Sep 24;11(10):1394. doi: 10.3390/biology11101394 (PMC9598447; doi:10.3390/biology11101394)
Supplement: Supplementary file 1 [file biology-11-01394-s001.zip › biology-1908534-supplementary.pdf]

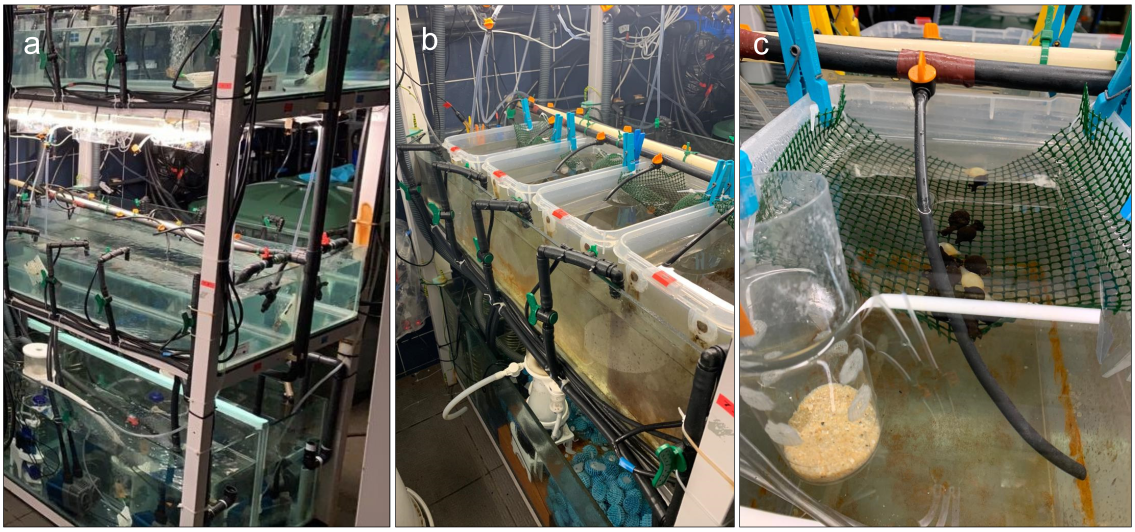

Figure S1. Aquaria system; (a) Recirculating system comprising two aquaria with two water-baths and a sump each, (b) four tanks in a bath (corresponding to a treatment); (c) cuttlefish eggs in a suspended net and cup containing a newborn cuttlefish.

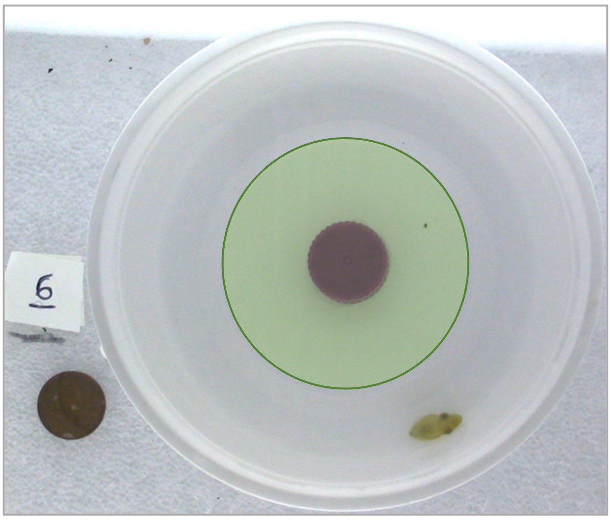

Figure S2. Photograph from a novel object test video frame. The purple cap is the novel object, and the green circle delineates the region of interest (in proximity to the object and slightly elevated).

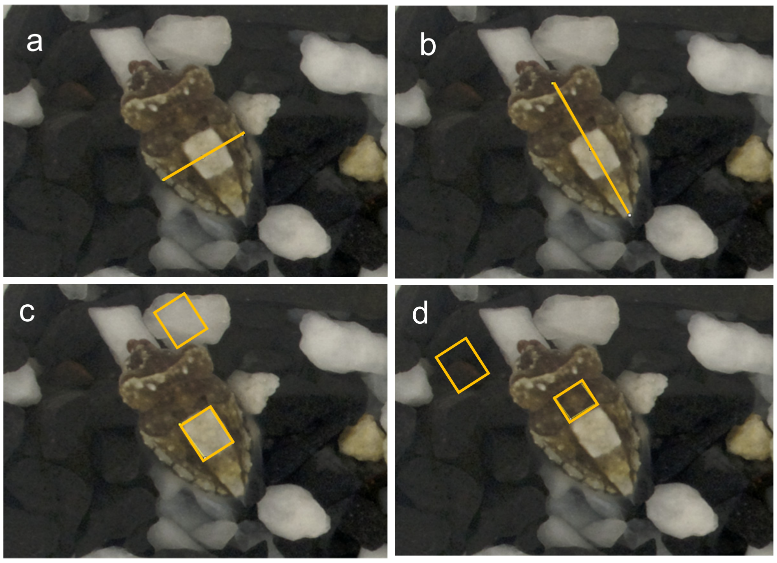

Figure S3. Metrics used to infer pixel intensity differences from photographs in the ImageJ software; (a) Cuttlefish transversal plane and (b) frontal plane used to measure pixel intensities; comparison of pixel integrated densities between (c) white substrate and cuttlefish light region and (d) black substrate and cuttlefish dark region.

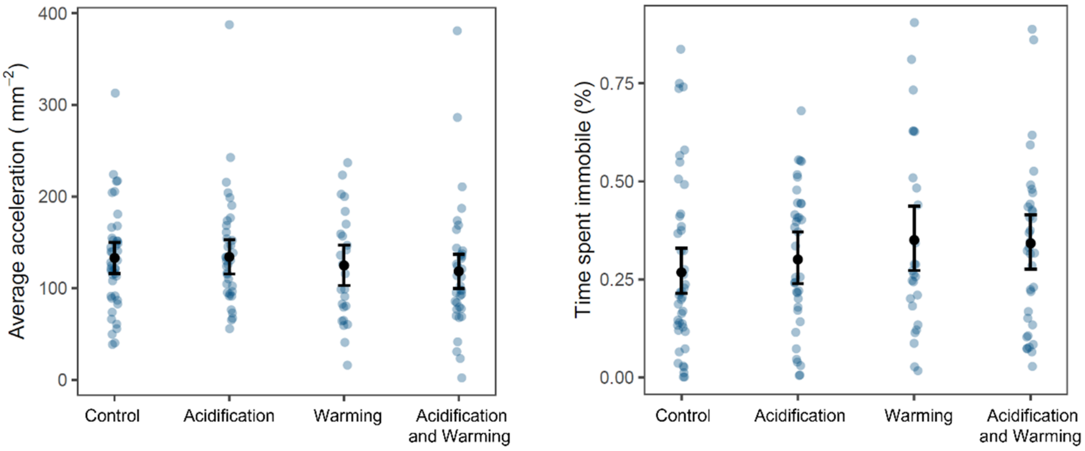

(**a**) (**b**)

Figure S4. Effect of treatments (control, acidification, warming, and acidification and warming) on cuttlefish (a) Average acceleration; (b) Percentage of time spent immobile. Points represent predicted means, and bars represent confidence intervals from generalized linear models (Gaussian and Beta families, respectively).

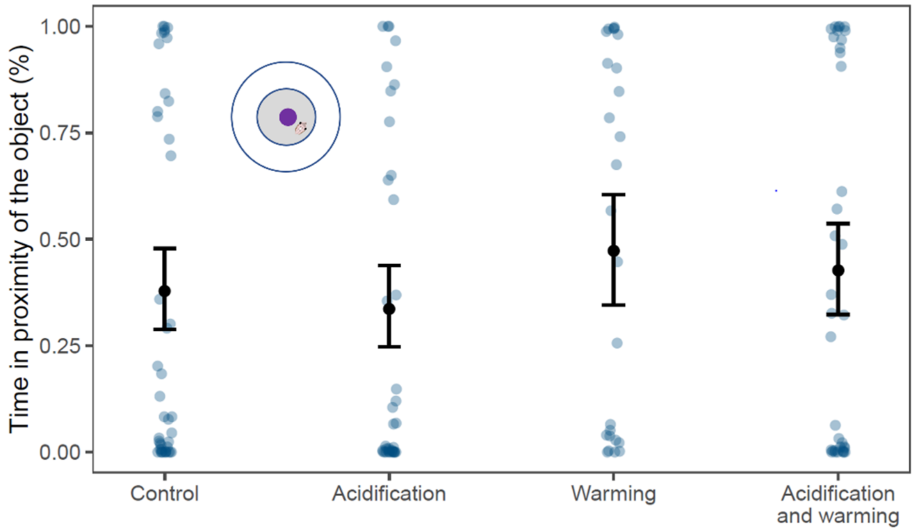

Figure S5. Effect of treatments (control, acidification, warming, and acidification and warming) on the percentage of time cuttlefish spent in proximity of the novel object. Points represent predicted means, and bars represent confidence intervals from a generalized linear model (Beta family). Blue points represent observed individual data.

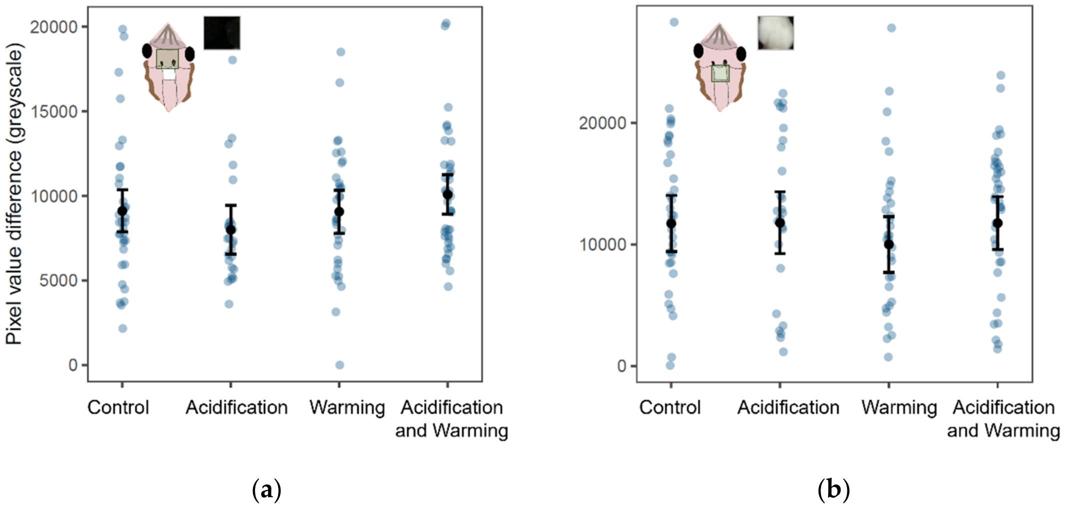

Figure S6. Pixel values range (difference between the maximum and minimum pixel intensity; grayscale units) between (a) Black gravel and the cuttlefish's dark region; (b) White gravel and the cuttlefish's light region (dorsal square) of cuttlefish, with relation to treatments: control, acidification, warming and acidification and warming combined. Points represent predicted means, and bars represent confidence intervals from linear models. Blue points represent observed individual data.

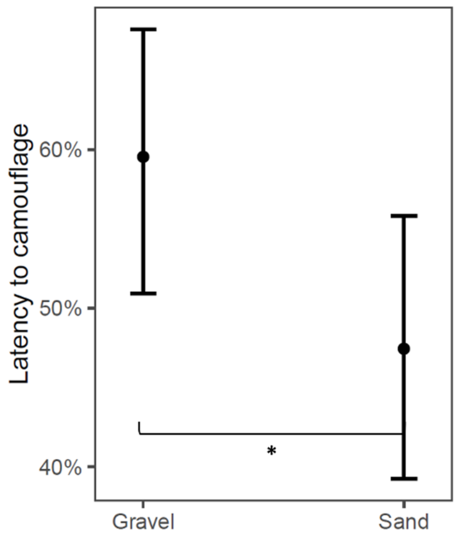

Figure S7. Influence of substrate (white and black gravel or sand) on the proportion of cuttlefish exhibiting delayed camouflage. Points represent predicted means, and whiskers represent confidence intervals from generalized linear models (Binomial and Poisson families, respectively).

Table S1. Seawater parameter values measured daily in each tank during exposure to treatments, shown as mean ± standard deviation. TA – Total Alkalinity, TCO2 – Total CO2, HCO3 - Bicarbonate, ΩAr – Aragonite saturation state.

Parameters	Control	Acidification	Warming	Combined
**Temperature**	18.2 ± 0.13	17.8 ± 0.07	20.8 ± 0.07	20.8 ± 0.08
**pH**	8.152 ± 0.08	7.726 ± 0.08	8.095 ± 0.06	7.737 ± 0.04
**Salinity**	34.0 ± 0.47	34.2 ± 0.46	33.7 ± 0.53	33.9 ± 0.74
**O2 (mg L^{-1})**	8.09 ± 0.10	8.10 ± 0.08	7.75 ± 0.11	7.71 ± 0.14
**O2 (%)**	105.9 ± 1.3	105.1 ± 1.0	106.4 ± 1.5	105.7 ± 1.8
**pCO_2 (μatm)**	321 ± 91	973 ± 165	374 ± 88	953 ± 106
**TA (μmol/kgSW)**	2457 ± 224	2460 ± 232	2454 ± 214	2472 ± 235
**TCO_2 (μmol/kgSW)**	2139 ± 210	2351 ± 211	2151 ± 206	2344 ± 219
**HCO_3 (μmol/kgSW)**	1899 ± 199	2217 ± 195	1919 ± 195	2201 ± 202
**ΩAr**	3.57 ± 0.62	1.55 ± 0.35	3.47 ± 0.42	1.75 ± 0.28

Table S2. Results from post-hoc multiple comparisons, depicting the effect of the treatments (control, acidification, warming, and acidification and warming combined) on the physiology, camouflage ability and anxiety parameters of *Sepia officinalis*.

Pairwise comparisons	Estimate	SE	z-ratio	*p*-value
**Mantle length (LM)**				
C – A	0.138	0.148	0.928	0.7898
C – W	-0.382	0.152	−2.508	0.0587
C – AW	-0.113	0.144	−0.785	0.8615
A – W	-0.244	0.153	−1.595	0.3816
A – AW	0.250	0.144	1.733	0.3066
W – AW	-0.494	0.148	−3.330	**0.0048**
**Survival over time (Cox model)**				
C – A	-	-	-	**5.3e-14**
C – W	-	-	-	**2.1e-15**
C – AW	-	-	-	**0.027**
A – W	-	-	-	**< 2e-16**
A – AW	-	-	-	**1.5e-08**
W – AW	-	-	-	0.064

**Latency to camouflage (gravel; Binomial GLM)**				
C – A	0.874	0.463	-0.253	0.9943
C – W	1.152	0.568	0.288	0.9901
C – AW	3.487	1.859	2.343	0.0912
A – W	1.008	0.536	0.015	1.0000
A – AW	0.251	0.143	-2.433	0.0733
W – AW	4.019	2.149	2.602	**0.0428**
**Pixel value difference in body planes (LM)**				
C – A	213	1340	0.159	0.9999
C – W	-4339	1246	-3.481	**0.0025**
C – AW	3290	1205	2.731	**0.0311**
A – W	-4125	1348	-3.061	**0.0081**
A – AW	-3077	1310	-2.348	0.0682
W – AW	-1049	1214	-0.864	0.8112

P-values in bold are inferior to 0.05.
